# The involvement of HPV in cervical oncogenesis

**DOI:** 10.1186/1471-2334-13-S1-O37

**Published:** 2013-12-16

**Authors:** Mihai Mitran, Carmen Georgescu, Sorin Puia, Loredana Mitran, Alexandra Bruja

**Affiliations:** 1 Clinical Hospital of Obstetrics and Gynecology “Prof. Dr. Panait Sârbu”, Bucharest, Romania; 2 Elias University Emergency Hospital, Bucharest, Romania

## Background

The site of action of HPV in the cervix is the squamous junction cylinder and the transformation zone. Abnormal epidermoid metaplasia and basal hyperplasia is the starting point for epithelial atypia.

## Methods

We assessed the dysplastic lesions of the cervix which underwent extended biopsy in the past 3 years - 579 cases and herpes virus type 2 infection was present in 2 cases.

## Results

HPV-HR (high-risk) was positive in 377 cases, HPV-LR (low-risk) was positive in 165 cases and in 35 cases no HPV typing was performed. The herpes virus type 2 infection was present in 2 cases.

Cytology showed LSIL (low grade squamous intraepithelial lesion) in 329 cases and HSIL (high grade squamous intraepithelial lesion) in 189 cases.

Histological examination revealed cervical intraepithelial neoplasia (CIN) I in 69 cases and CIN II, CIN III in 183 cases and carcinoma in situ in 6 cases.

HSIL cases all presented infection with HR (16, 18, 31) strains.

## Conclusion

Concomitance of cervical dysplasia with HSIL cytology is a major risk factor in cervical neoplasia.

